# Small-molecule inhibition of APE1 induces apoptosis, pyroptosis, and necroptosis in non-small cell lung cancer

**DOI:** 10.1038/s41419-021-03804-7

**Published:** 2021-05-18

**Authors:** Kaili Long, Lili Gu, Lulu Li, Ziyu Zhang, Enjie Li, Yilan Zhang, Lingfeng He, Feiyan Pan, Zhigang Guo, Zhigang Hu

**Affiliations:** Jiangsu Key Laboratory for Molecular and Medical Biotechnology, College of Life Sciences, Nanjing Normal University, 1 WenYuan Road, 210023 Nanjing, China

**Keywords:** Non-small-cell lung cancer, Apoptosis

## Abstract

Apurinic/apyrimidinic endonuclease 1 (APE1) plays a critical role in the base excision repair (BER) pathway, which is responsible for the excision of apurinic sites (AP sites). In non-small cell lung cancer (NSCLC), APE1 is highly expressed and associated with poor patient prognosis. The suppression of APE1 could lead to the accumulation of unrepaired DNA damage in cells. Therefore, APE1 is viewed as an important marker of malignant tumors and could serve as a potent target for the development of antitumor drugs. In this study, we performed a high-throughput virtual screening of a small-molecule library using the three-dimensional structure of APE1 protein. Using the AP site cleavage assay and a cell survival assay, we identified a small molecular compound, NO.0449-0145, to act as an APE1 inhibitor. Treatment with NO.0449-0145 induced DNA damage, apoptosis, pyroptosis, and necroptosis in the NSCLC cell lines A549 and NCI-H460. This inhibitor was also able to impede cancer progression in an NCI-H460 mouse model. Moreover, NO.0449-0145 overcame both cisplatin- and erlotinib-resistance in NSCLC cell lines. These findings underscore the importance of APE1 as a therapeutic target in NSCLC and offer a paradigm for the development of small-molecule drugs that target key DNA repair proteins for the treatment of NSCLC and other cancers.

## Introduction

Globally, lung cancer is the most common cancer type and is the leading cause of cancer-related death^1^. Non-small cell lung cancer (NSCLC) accounts for ~85% of all lung cancer cases and can be pathologically categorized into two major histological types: lung adenocarcinoma (LUAD) and lung squamous cell carcinoma (LUSC)^2^. Defects in cell cycle checkpoints and DNA damage/repair capacity might contribute to elevated lung cancer risks^3^. Patients with lung cancer have been associated with lower DNA repair capacities and higher rates of spontaneous and carcinogen-induced chromosomal aberrations than healthy controls^3,4^. An elevated DNA repair capacity in cancer cells is thought to contribute to the development of resistance against radiation and platinum-based chemotherapy^5,6^. For example, the expression and activity of 8-oxoguanine DNA N-glycosylase (OGG1), which catalyzes the first step in the DNA base excision repair (BER) pathway, have been associated with the development of resistance against platinum-based chemotherapy in NSCLC^4,7^. Our previous study indicated that the overexpression of another key BER pathway enzyme, flap structure-specific endonuclease 1 (FEN1), promoted tumor progression and conferred cisplatin (DDP) resistance in NSCLC^8^. Other BER pathway enzymes and proteins, such as apurinic/apyrimidinic endonuclease 1 (APE1), DNA polymerase β (pol β), and X-ray repair cross-complementing 1 (XRCC1), have been associated with.

NSCLC development and the acquired resistance of cancer cells against chemotherapy and radiotherapy^7,9,10^. These studies have implied that the BER pathway plays a key role in NSCLC development and therapy.

APE1, a key enzyme in the BER system, plays a pivotal role in the repair of oxidized and alkylated genomic DNA bases by identifying and cleaving nucleotide chains at 5’ apurinic (AP) sites^11^. APE1 cleaves the phosphodiester bonds at an AP site by orienting the cleavage site in an optimal position for a nucleophilic attack within its compact active site, leaving 3′-hydroxyl and 5′-deoxyribose phosphate (d RP) groups flanking the nucleotide gap^12^. In addition to AP endonuclease activity, APE1 exhibits 3′–5′ exonuclease, 3′-phosphodiesterase, 3′ RNA phosphatase, and 3′ exoribonuclease activities, which allows APE1 to act on a wide variety of DNA and RNA substrates^12^. These APE1 activities depend on its C-terminal DNA repair domain, whereas the N-terminus is essential for the redox activity attributed to APE1^11,12^. APE1 has been independently identified to act as a reductive activator, stimulating several transcription factors (for this reason, APE1 is also known as redox effector factor 1, ref. ^1^), including signal transducer and activator of transcription 3 (STAT3), activating transcription factors (ATF)/cAMP response element-binding protein (CREB), nuclear factor-kappa B (NF-κB), paired box 5 (PAX5), PAX8, activator protein 1 (AP−1), hypoxia-inducible factor (HIF)-1α, p53, c-Myb, and others^11,13^. APE1 has been shown to interact with nucleophosmin (NPM1), a protein involved in ribosomal RNA (rRNA) biogenesis, which regulates the subcellular localization and endonuclease activity of APE1^14^. These findings implicate APE1 in RNA metabolism, cell proliferation, and cancer progression, as well as neurodegenerative disease^12,15^.

Recently, studies examining the relationship between APE1 and cancer pathology have emerged. The elevated expression of APE1 has been detected in many types of cancers, including breast cancer, lung cancer, bladder cancer, ovarian cancer, and pancreatic cancer^16–20^. The inhibition of APE1 expression or activity impacts the cell cycle, cell proliferation, colony formation, and apoptosis of cancer cells^20,21^. The blockade of APE1 activity by inhibitors has been demonstrated to induce lethality in breast cancer susceptibility (BRCA)- and ataxia-telangiectasia mutated (ATM)-deficient cells^22^. Moreover, APE1 overexpression has been associated with the increased resistance against chemotherapeutic drugs and radiation in several cancer types^10,23^. Previous studies have shown that APE1 activity confers resistance against radiation and chemotherapy in medulloblastoma cells^24^. APE1 can also increase the resistance of human glioma cells against alkylating agents^25^. For these reasons, APE1 is considered to be a promising prognostic cancer biomarker and therapeutic target.

Recently, emerging studies have described the use of APE1 inhibitors for cancer therapy. Low-molecular concentrations of the selective APE1 inhibitor CRT0044876 inhibited the AP endonuclease, 3’-phosphodiesterase, and 3’-phosphatase activities of APE1 and improved the sensitivity of HT1080 cells to methylmethane sulfonate (MMS) and temozolomide (TMZ)^26^. AJAY-4, a nanomolar APE1 inhibitor, has been shown to enhance the abasic sites that cleave and activate poly (ADP-ribose) polymerase (PARP), resulting in the activation of caspase-3 and caspase-7 and subsequent NCI-60 cell death by apoptosis^27^. Another more widely studied APE1 inhibitor, E3330/APX3330, has been identified as a direct and highly specific inhibitor of the APE1-redox function^28^. APX3330 impaired tumor growth and blocked the activities of transcription factors, such as NF-κB, AP-1, HIF-1α, and STAT3. At the molecular level, APX3330 increases the formation of disulfide bonds between C65 or C93 residues in APE1, which are involved in the redox regulation of AP-1^11,29^. Recently, APX3330 has entered Phase 1 anticancer clinical trials, with demonstrated safety, efficacy, and APE1 target engagement^29,30^. A second-generation APE1-targeting molecule, APX2009, has been described as an effective small molecule that is neuroprotective against cisplatin- and oxaliplatin-induced toxicity^30^.

Due to the functional significance of APE1 in NSCLC progression, the inhibition of APE1 represents a potential strategy for NSCLC therapy. The knockdown of APE1 by small interfering RNA (siRNA) inhibited X-ray irradiation-induced angiogenesis in A549 cells^31^. Adenovirus-mediated APE1 silencing enhanced the tumor suppression efficacy of photodynamic therapy in an A549 xenograft model^32^. APX3330 treatment significantly enhanced the tyrosine kinase inhibitor (TKI)-induced inhibition of cell growth and enhanced apoptosis in TKI-resistant LUAD cells, whereas treatment with an APE1 DNA repair activity inhibitor, APE1 inhibitor III, showed no effect^33^. Furthermore, APX3330 treatment effectively reversed the epithelial‐to‐mesenchymal transition (EMT) phenotype and further sensitized cells to epidermal growth factor receptor (EGFR)‐TKIs^34^. In addition, APX3330 increased the cisplatin-induced cytotoxicity and impairment of cell migration and invasion in NSCLC cells, indicating that APX3330 might represent a promising compound for boosting cisplatin therapy^17^. However, the application of APE1 endonuclease inhibitors for NSCLC therapy remains limited. In this study, we identified NO.0449-0145 as a novel APE1 endonuclease activity inhibitor using molecular docking and biochemical analyses. We further identified that NO.0449-0145 induces apoptosis, pyroptosis, and necroptosis in NSCLC cells. Furthermore, NO.0449-0145 also efficiently suppressed the proliferation of cisplatin-resistant A549-DDP cells and erlotinib-resistant NCI-H1975 cells by inducing apoptosis, pyroptosis, and necroptosis.

## Results

### APE1 is overexpressed in NSCLCs and correlates with malignancy

**Fig. 1:**
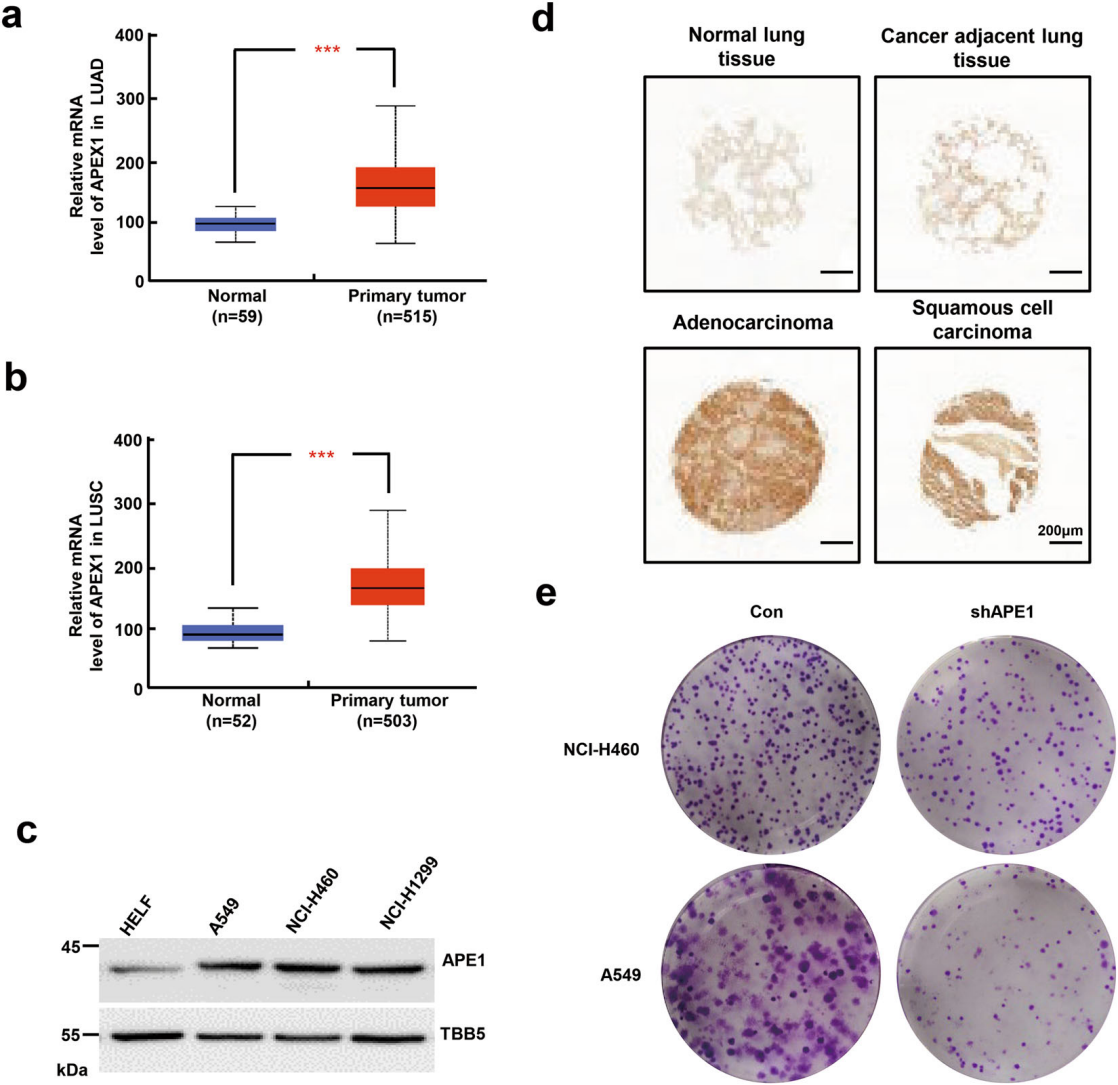
Expression levels of APE1 are positively correlated with NSCLC. **a**, **b** Statistical analysis of APE1 mRNA expression levels in LUAD and LUSC from the TCGA database. **c** APE1 protein levels in normal lung HELF cell, NSCLC cell lines A549, NCI-H460, and NCI-H1299. **d** Immunohistochemical staining of APE1 in labeled normal lung tissues or tumor tissues. **e** Colony-formation assay in control or APE1-KD A549 and NCI-H460 cells. Representative images from one of three experiments are shown. Data are shown as the mean ± SD of three independent experiments. ***p < 0.001.

According to data retrieved from the Cancer Genome Atlas (TCGA) database, APE1 mRNA expression levels were significantly higher in both LUAD and LUSC tumor tissues compared with those in normal tissues (Fig.1a and b). In addition, APE1 protein and mRNA expression levels were upregulated in three NSCLC cell lines, A549, NCI-H460, and NCI-H1299, compared with those in the normal human lung fibroblast (HELF) cells (Fig. 1c and Supplementary Fig. 1a). We employed a tissue array (LC1005a, Avilabio) to explore APE1 expression levels among NSCLC specimens and normal tissues. We found that APE1 was highly expressed in both adenocarcinoma and squamous cell carcinoma tissues compared with adjacent and normal lung tissues (Fig. 1d). These results were consistent with the data obtained from the TCGA database. The colony-formation assay showed that A549 and NCI-H460 proliferation was suppressed following APE1 knockdown (Fig. 1e and Supplementary Fig. 1b). Consistently, a previous study demonstrated that the median overall survival time and overall survival of NSCLC patients with high-expression APE1 were reduced compared with those of a low APE1 expression group^23^. These results suggested that APE1 is overexpressed in NSCLC and associated with malignancy.

### Screening of small-molecule APE1-inhibiting compounds

**Fig. 2:**
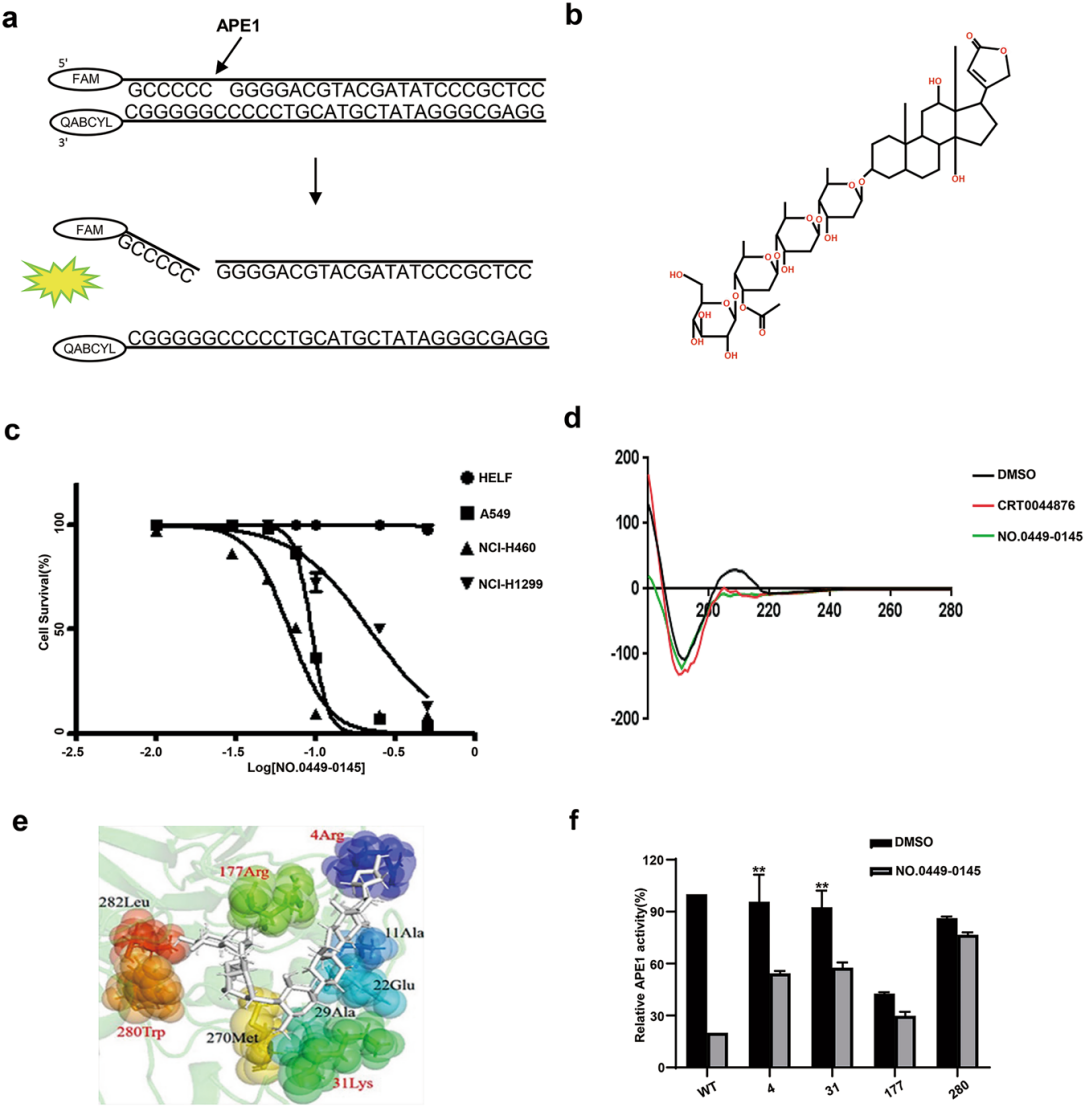
Screening of small molecular inhibitor of APE1. **a** The schemata of AP site cleavage assay. **b** Chemical structure of NO.0449-0145. **c** Cell survival assay showed that NO.0449-0145 treatment induced cell death in A549, NCI-H460, NCI-H1299 cells but not in HELF cell. **d** Circular dichroism spectroscopy analysis of APE1/NO.0449-0145 binding. **e** Docking studies identified four high-potential sites of APE1 binding with NO.0449-0145. **f** Inhibitory effects of NO.0449-0145 on FEN1 mutations. Each experiment was repeated three times. **p < 0.01.

We performed a high-throughput virtual screening of a small-molecule library (ChemDiv library) using the three-dimensional structure of APE1. The four best druggable sites of the APE1 protein were predicted using a druggable site prediction (DSP) algorithm (Supplementary Fig. 2a)^35^. The molecular docking study was used to calculate and predict the potential interaction affinity between small-molecule compounds and the APE1 DNA repair active site. Using this method, we identified 52 small molecular compounds as potential APE1 inhibitors (Supplementary Table 1). These 52 small molecular compounds were then tested for their effects on the modulation of APE1 endonuclease activity using the AP site cleavage assay (Fig. 2a). In the AP site cleavage assay, fluorescence intensity increases when APE1 cleaves the AP site on the DNA substrate, and the addition of an effective inhibitor represses the rate of increase in fluorescence intensity (Supplementary Fig. 2b). Eight compounds were identified with stronger inhibitory effects than the positive control, CRT0044876, which was previously reported to inhibit APE1 endonuclease activity. The vehicle, dimethyl sulfoxide (DMSO), was used as the negative control (Supplementary Table 2). To identify the effects of these eight potential APE1 inhibitors in cells, a cell survival assay was performed in A549 cells. Among the eight tested compounds, NO.0449-0145 showed the most effective inhibitory activity with a half-maximal inhibitory concentration (IC50) of 0.1068 µM in A549 cells (Fig. 2b and Supplementary Table 3). A cell survival assay was then performed in A549, NCI-H460, NCI-H1299, and HELF cells to examine the inhibitory effects of NO.0449-0145 in other NSCLC cell lines and control HELF cells. We found that this inhibitor significantly inhibited cell survival in NSCLC cells but not HELF cells, which express low levels of APE1 (Fig. 2c). In contrast, the previously reported APE1 endonuclease inhibitor CRT0044876 showed no cytotoxic effects in either A549 or NCI-H460 cells even at high concentrations (Supplementary Fig. 3).

The molecular simulation assay examining the structures of APE1 and NO.0449-0145 indicated that NO.0449-0145 could directly interact with APE1 (Supplementary Fig. 2c). To confirm the binding of NO.0449-0145 with APE1, we performed a circular dichroism spectroscopy analysis. The results showed a clear shift in the spectrum upon the addition of NO.0449-0145 to APE1, indicating a direct physical interaction between the small molecule and APE1 (Fig. 2d). This result was consistent with the results of the molecular simulation analysis. Furthermore, we examined which APE1 sites were critical for this interaction by performing docking studies, which identified four high-potential sites for the binding of APE1 with NO.0448-0145: R4, K31, R177, and W280 (Fig. 2e). To further validate whether R4, K31, R177, and W280 were involved in the NO.0449-0145/APE1 interaction, we generated the following APE1 mutations: R4G, K31G, R177G, and W280G. The AP site cleavage assay performed using these APE1 mutant proteins showed that although the AP site endonuclease activity of the mutants R4G, K31G, and W280G remained mostly intact, the inhibitory effects of NO.0449-0145 were disrupted, at least partially, for these mutants (Fig. 2f). The AP site endonuclease activity of the R177G mutant was almost completely abolished because this residue is essential for substrate binding during DNA repair by APE1^36,37^. These results suggested that these residues are involved in the NO.0449-0145-related inhibition of APE1 endonuclease activity. Furthermore, electrophoresis mobility shift assay (EMSA) was employed to investigate the effect of NO.0449-0145 on the redox activity of APE1. Our results showed that NO.0449-0145 has no effect on the redox activity of APE1 (Supplementary Fig. 4).

### NO.0449-0145 induced DNA damage in NCI-H460 and A549 cells

**Fig. 3:**
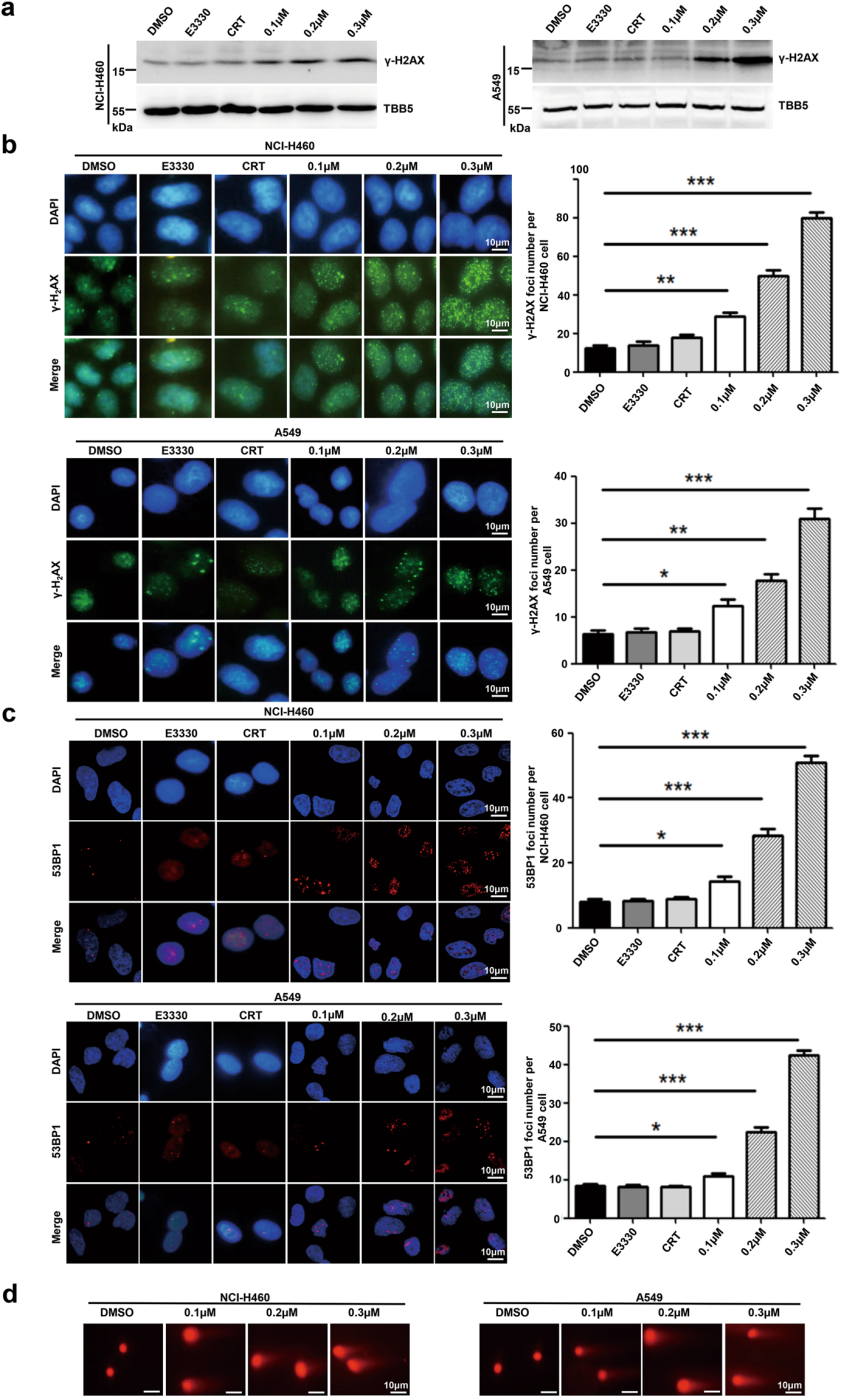
Inhibition of FEN1 by NO.0449-0145 leads to the accumulation of DNA damage. **a** Western blot assay to determine γ-H2AX levels in A549 and NCI-H460 cells following different dose of NO.0449-0145 treatments. **b**, **c** Immunofluorescence staining of γ-H2AX foci and 53BP1 foci in cells. The quantification of the foci numbers per cells were showed in right panel. **d** Comet assay of A549 and NCI-H460 cells treated with or without NO.0449-0145. Representative images for the quantification of the average tail moment reported for each treatment condition. Each experiment was repeated three times. *p < 0.05; **p < 0.01; ***p < 0.001.

APE1 is essential for the BER pathway; therefore, APE-deficient cells could accumulate AP sites, triggering DNA damage and leading to cell death. Therefore, we detected the levels of DNA damage in both NCI-H460 and A549 cells following NO.0449-0145 treatment. As shown in Fig. 3a, the expression levels of the phosphorylated histone γ-H2AX were elevated in both NCI-H460 and A549 cells treated with NO.0449-0145. γ-H2AX- and p53-binding protein 1 (53BP1)-positive foci were detected in NCI-H460 and A549 cells treated with NO.0449-0145 by immunofluorescence assay. We observed that NO.0449-0145 treatment increased the foci numbers of γ-H2AX and 53BP1 compared with control conditions in both cell lines (Fig. 3b, c). Additionally, the alkaline comet assay, which is frequently used to identify single-stranded breaks (SSBs), revealed that NO.0449-0145 treatment increased the levels of spontaneous DNA strand breaks compared with vehicle (Fig. 3d). In contrast, NO.0449-0145 treatment showed no effect on the accumulation of DNA damage in HELF cells (Supplementary Fig. 5), which was consistent with results showing that NO.0449-0145 repressed NSCLC cells but not HELF cells. These results suggested that NO.0449-0145 treatment induced the accumulation of DNA damage in both NCI-H460 and A549 cells.

### NO.0449-0145 induces cell apoptosis, pyroptosis, and necroptosis

**Fig. 4:**
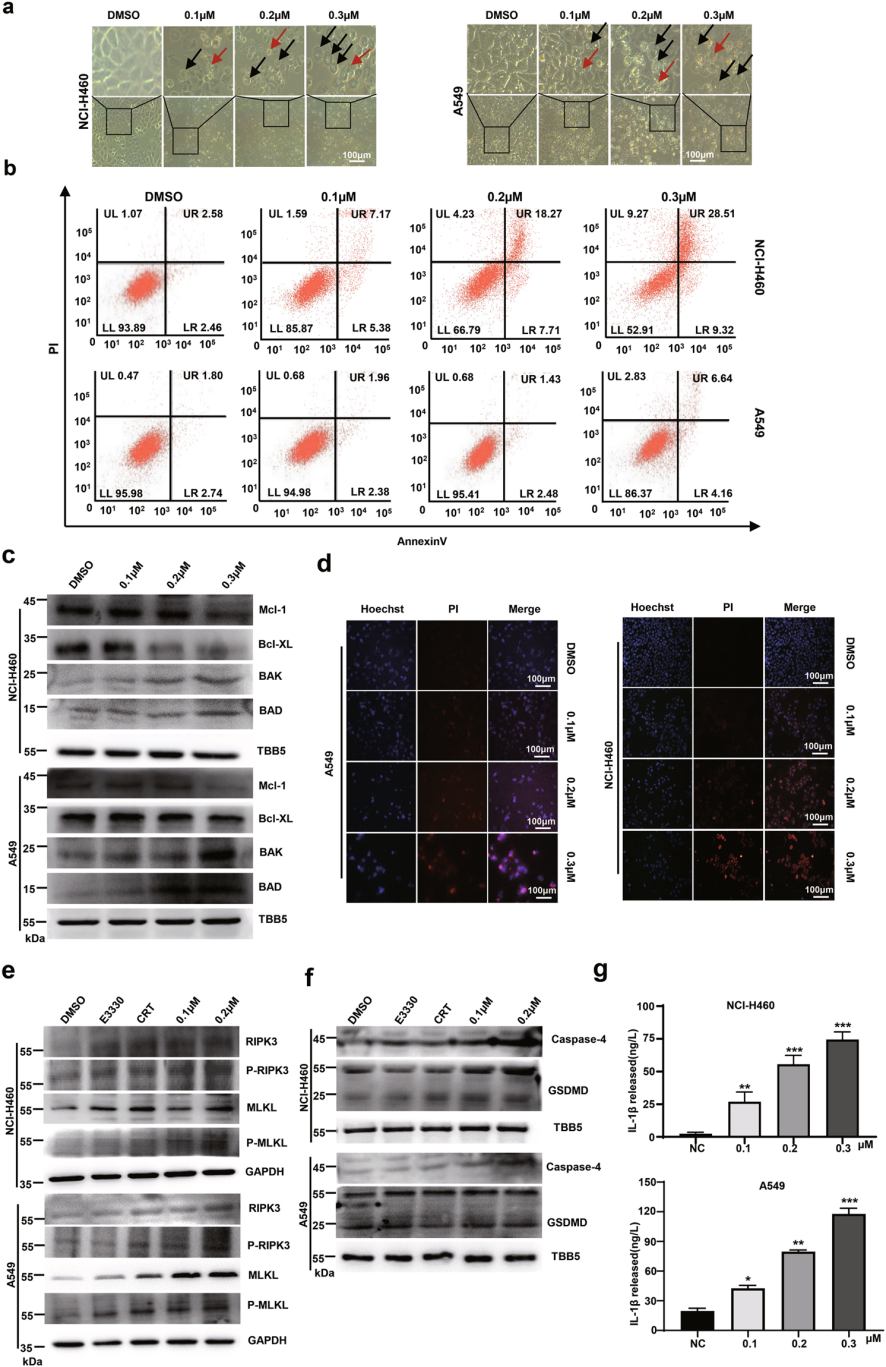
NO.0449-0145 induces cell death in NCI-H460 and A549 cells. **a** Morphological analysis of NCI-H460 and A549 cells with different dose of NO.0449-0145 treatment for 24 h. The red arrows indicate the apoptotic bodies; the black arrows point to bubbling of cells. **b** Annexin V/PI staining and flow cytometry analysis of NCI-H460 and A549 cells with different dose of NO.0449-0145 treatment for 24 h. **c** Levels of BAD, BAK, Mcl-1 and Bcl-XL assessed by western blotting for the cells treated with NO.0449-0145 for 12 h. **d** Representative Hoechst/PI fluorescence images of cells with different treatment. **e** Western blotting analysis of basic RIPK3, MLKL, phosphorylation of RIPK3 (P-RIPK3), and MLKL (P-MLKL) in A549 and NCI-H460 cells. **f** Western blotting analysis of caspase-4 and GSDMD in A549 and NCI-H460 cells. **g** Secreted IL-1β from cells treated with NO.0449-0145 for 24 h. Each experiment was repeated three times. The significance between treated samples and control samples was determined using a *t-*test. *p < 0.05; **p < 0.01; ***p < 0.001.

A cell survival assay demonstrated that NO.0449-0145 could repress NCI-H460 and A549 cell survival at a very low IC50 (Fig. 2c). These results were verified by a morphological analysis, which showed that NO.0449-0145 treatment induced cell death in both NCI-H460 and A549 cells in a dose-dependent manner (Fig. 4a). The patterns associated with NO.0449-0145-induced cell death were analyzed with microscopic imaging. As expected, both cell types displayed normal features upon exposure to vehicle, whereas NO.0449-0145-treated cells showed various cell morphologies. Microscopic imaging showed that some cells shrank and became dense, featuring classic apoptotic bodies. In contrast, some cells featured cytoplasmic swelling and plasma membrane bubbles (Fig. 4a). These microscopic imagines were suggestive of cells undergoing apoptosis, pyroptosis, or necroptosis following NO.0449-0145 treatment.

To verify whether NO.0449-0145 induced apoptosis, we performed Annexin V-fluorescein isothiocyanate (FITC)/propidium iodide (PI) staining, which is frequently used to measure apoptosis. As shown in Fig. 4b, NO.0449-0145 treatment for 24 h induced apoptosis (Q2 late apoptosis and Q4 early apoptosis) in NCI-H460 cells in a concentration-dependent manner compared with vehicle treatment. A similar result was observed in A549 cells. Western blot assay was used to detect whether NO.0449-0145 alters the expression levels of anti- and pro-apoptotic proteins in NCI-H460 and A549 cells. The Bcl-2 family, which consists of pro-apoptotic proteins (such as BAD and BAK) and anti-apoptotic proteins (such as Mcl-1 and Bcl-XL), plays a vital role in the regulation of cell apoptosis. Our data showed that Bcl-XL and Mcl-1 were downregulated, whereas BAD and BAK were upregulated in a NO.0449-0145 dose-dependent manner in NCI-H460 cells treated for 12 h (Fig. 4c), and a similar result was observed in A549 cells. We also detected changes in the mitochondrial membrane potential (MMP) using JC-1 staining and flow cytometry. The results showed that the MMP decreased in NCI-H460 cells treated with NO.0449-0145 for 24 h, whereas only a slight decrease in the MMP was detected in A549 cells treated with the same dose of NO.0449-0145 (Supplementary Fig. 6). These results collectively indicated that treatment with NO.0449-0145 potently induced cell apoptosis in NSCLC cells.

In addition, the morphological analysis revealed cytoplasmic swelling and plasma membrane bubbles in NCI-H460 and A549 cells treated with NO.0449-0145, indicating that these cells were likely to be undergoing pyroptosis or necroptosis^38,39^. We further examined cells using Hoechst33342/PI staining. Hoechst33342 can penetrate the cell membrane and bind DNA, and the fluorescence of apoptotic cells becomes significantly enhanced compared with that of normal cells. In contrast, PI cannot penetrate the cell membrane and cannot stain normal cells or apoptotic cells with intact cell membranes. During necrosis, the integrity of the cell membrane is lost, allowing PI to enter and stain necrotic cells. Our data showed that PI-positive cells increased in NO-0449-0145 dose-dependent (Fig. 4d) and time-dependent manners (Supplementary Fig. 7a) in both NCI-H460 and A549 cells following NO.0449-0145 treatment. The phosphorylation of both receptor-interacting serine/threonine kinase 3 (RIPK3) and mixed lineage kinase domain-like pseudokinase (MLKL), which have emerged as key cellular components during necroptosis^40^, increased in both NCI-H460 and A549 cells treated with NO.0449-0145 for 12 h (Fig. 4e). Furthermore, the protein levels of caspase-4 and cleaved gasdermin D (GSDMD), which play key roles in cell pyroptosis increased following NO.0449-0145 treatment for 12 h (Fig. 4f)^41^, which paralleled the observation that the cellular and secreted pro-inflammatory cytokines IL-1βwere upregulated (Fig. 4g and Supplementary Fig. 7b).

### NO.0449-0145 suppresses NSCLC xenograft tumors

**Fig. 5:**
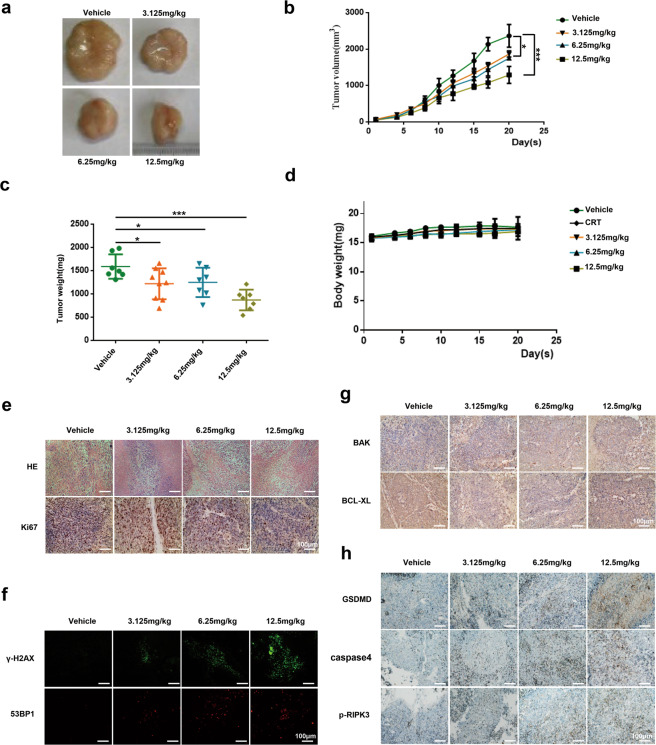
NO.0449-0145 suppresses NSCLC xenograft tumors. **a**–**c** Tumor volumes and weights monitored from mice (*n* = 5 per group) treated with vehicle or different dose of NO.0449-0145 (3.125, 6.25, and 12.5 mg/kg) every 2 days. **d** Mice body weights monitored during treatment. **e** Representative micrographs of H&E staining and IHC staining for Ki67. **f** Representative micrographs of Immunofluorescence staining for γ-H2AX and 53BP1 foci. **g**, **h** Representative micrographs of IHC staining for BAK, BCL-XL, GSDMD, caspase-4, RIPK3, and phosph-RIPK3. The significance between treated samples and control samples was determined using a *t-*test. *p < 0.05; ***p < 0.001.

Next, we assessed the antitumor effects of NO.0449-0145 in vivo, using nude mice to perform a xenograft study. NCI-H460 cells were subcutaneously implanted into the hind flanks of each nude mouse, and the mice were randomized into four groups (5 mice per group) when the tumors reached 100 mm^3^. The mice were then intraperitoneally injected with either NO.0449-0145 (3.125, 6.25, or 12.5 mg/kg, one quarter of the median lethal dose [50 mg/kg] or much less dose were used) or vehicle (saline) with volume of 100 µl every two days. Our data showed that treatment with NO.0449-0145 suppressed tumor growth in a dose-dependent manner, with both the final tumor volumes and weights decreasing following NO.0449-0145 treatment (Fig. 5a-c). No obvious toxicity was observed in any of the treatment groups, based on average body weight and size and morphology of spleen and kidney, which did not differ significantly from that of control mice (Fig. 5d and Supplementary Fig. 8). These results suggested that NO.0449-0145 could suppress xenograft tumor growth in vivo.

To investigate the in vivo mechanism of antitumor action for NO.0449-0145, an immunohistochemistry/immunofluorescence assay was conducted using NCI-H460 tumor samples collected from the various treatment groups. As shown in Fig. 5e, f, NO.0449-0145 treatment increased the positive staining for γ-H2AX and 53BP1 in tumor tissues and impaired the expression of Ki67, which is a known marker of cell proliferation. We further detected the expression levels of BAK and Bcl-XL in tumor tissues and found a dose-dependent increase in BAK expression and decrease in Bcl-XL expression in NO.0448-0145-treated tumor tissues (Fig. 5g). Additionally, the immunohistochemistry assay identified the upregulation of caspase-4, GSDMD, and phospho-RIPK3 expression in xenograft tumor tissues following NO.0449-0145 treatment (Fig. 5h). These results were consistent with the results of our cell-based experiments. Taken together, these findings suggested that NO.0449-0145 treatment suppressed xenograft growth in nude mice by aggravating DNA damage, inhibiting cell proliferation, and inducing cell death in tumor cells.

### NO.0449-0145 overcomes the cisplatin resistance of NSCLC cells

**Fig. 6:**
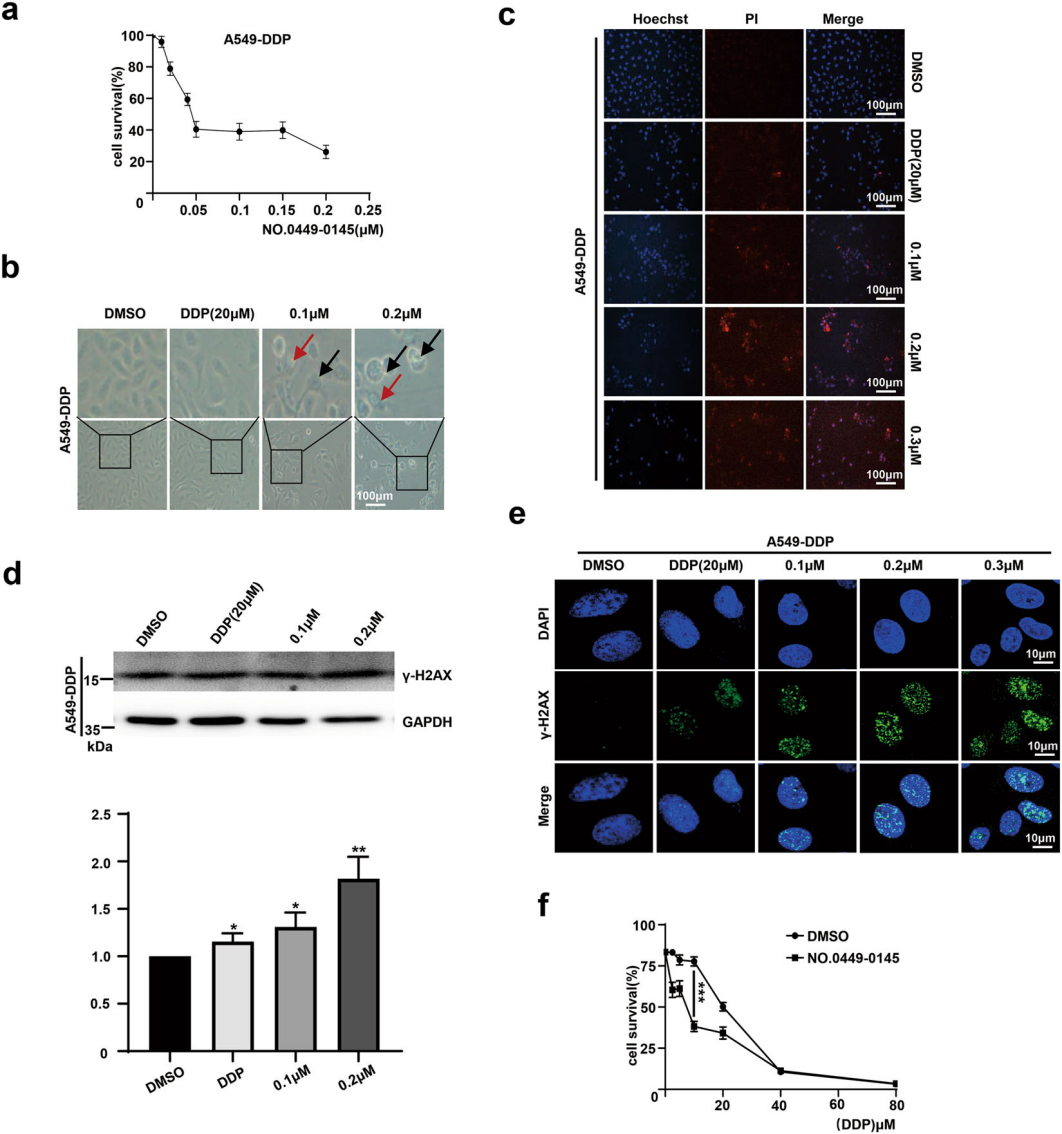
NO.0449-0145 overcomes the cisplatin resistance of A549 cell. **a** Cell survival assay in A549-DDP cells treated with NO.0449-0145 for 48 h. **b** Morphological analysis of different treated A549-DDP cells. **c** Representative Hoechst/PI fluorescence images of A549-DDP cells with different treatment. **d** Western blotting analysis of γ-H2AX in different treated A549-DDP cells. **e** Representative image of Immunofluorescence staining for γ-H2AX foci. **f** Drug sensitivity assay of A549-DDP cells following co-treatment with cisplatin and NO.0449-0145 or vehicle. The data are presented as the mean ± SD values, and the error bars represent data from triplicate biological experiments. *p < 0.05; **p < 0.01; ***p < 0.001.

Cisplatin is one of the first-line chemotherapeutic drugs for used NSCLC treatment^5^. After uptake into cells, cisplatin binds to DNA and forms various types of DNA adduct, blocking DNA replication and inducing DNA damage, which eventually triggers apoptosis or necrosis^5^. However, the increased DNA repair efficiency in cancer cells can result in the development of drug resistance, limiting the efficacy of chemotherapeutic drugs^42^. Thus, we evaluated the effects of NO.0449-0145 on cisplatin (DDP)-resistant A549 (A549-DDP) cells. A cell survival assay indicated that NO.0449-0145 treatment markedly induced cell death in A549-DDP cells (Fig. 6a and Supplementary Fig. 9). These results were verified by a morphological analysis, which revealed cells with apoptotic bodies, cytoplasmic swelling, and plasma membrane bubbles (Fig. 6b), similar to those observed in Fig. 4. The Hoechst33342/PI staining indicated that plasma membrane permeabilization increased in a dose-dependent manner following NO.0449-0145 treatment (Fig. 6c). These results indicated that NO.0449-0145 treatment induced cell apoptosis, pyroptosis, and necroptosis in A549-DDP cells. Furthermore, the expression levels and foci containing γ-H2AX were upregulated in A549-DDP cells after NO.0449-0145 treatment (Fig. 6d, e), indicating the elevation of DNA damage levels in NO.0449-0145-treated cells. A cell survival assay, using various combinations of NO.0449-0145 treatment and different doses of cisplatin, indicated that treatment with NO.0449-0145 was able to overcome DDP-resistance in A549-DDP cells (Fig. 6f).

## Discussion

As a key enzyme in the BER pathway, APE1 catalyzes the cleavage of the DNA phosphodiester backbone on the 5′ side of an AP site, generating a nick in the DNA, which can then be processed and replaced by one (short patch) or more (long patch) nucleotides mediated by a series of protein complexes^29,43^. APE1 has been identified as a biomarker and potential therapeutic target in several types of cancers. APE1 contains two major domains, a redox domain and a DNA repair domain, which correspond with redox activity and endonuclease activity, respectively. APE1 inhibitors can, therefore, be designed to disrupt either of these two activities. To date, however, only inhibitors targeting the APE1-redox activity have demonstrated therapeutic efficacy in cancer cell lines^29^. Recently, APX3330 became the first APE1-redox inhibitor to complete Phase I clinical trials for cancer treatment^44^. Due to the important role played by APE1 in DNA repair, identifying an APE1 inhibitor that targets the DNA repair activity of APE1 is likely to represent a critical strategy for tumor therapy. Here, we identified 52 potential APE1-binding compounds from a small-molecule library (ChemDiv library) through a structure-based virtual screening technique and further verified the effects of the potent APE1 inhibitory molecule NO.0449-0145 by performing AP site cleavage and cell survival assays. Molecular simulation analysis and circular dichroism spectroscopy analysis confirmed the binding between NO.0449-0145 and APE1. The cell survival assay identified the repressive effects of NO.0449-0145 on the proliferation of various NSCLC cell lines, and a xenograft study further confirmed that NO.0449-0145 repressed NSCLC cells both in vitro and in vivo.

As expected, the inhibition of APE1 by NO.0449-0145 induced the accumulation of DNA damage in both A549 and NCI-H460 cells, which was consistent with the hypothesis that cancer cells would accumulate DNA damage when treated with inhibitors that target the DNA repair pathway^45^. In contrast, the accumulation of γ-H2AX and recruitment of 53BP1 were reduced in cells treated with the APE1-redox inhibitor APX3330 or with the previously reported APE1 endonuclease inhibitor CRT0044876, which demonstrated no cytotoxic effects in NSCLC cells, even at high concentrations. The alkaline comet assay confirmed the accumulation of SSBs and double-stranded breaks in A549 and NCI-H460 cells following treatments with NO.0449-0145, resulting in the accumulation of DNA fragmentation. This increase in DNA damage and fragmentation might further induce cell death through apoptosis, pyroptosis, and necroptosis^6,45–47^.

Cells can be subjected to accidental cell death (ACD) and regulated cell death (RCD) under both normal and abnormal conditions. ACD occurs in response to severe physical, chemical, or mechanical insults (e.g., high pressure and high temperature) and is typically independent of molecular signaling pathways^40,47^. In contrast, RCD originates from the activation of an endogenous physiological program, often due to perturbations in the intracellular or extracellular microenvironment that are too intense or prolonged for adaptive responses, which fail to restore cellular homeostasis^40,48^. RCD involves tightly structured signaling cascades and molecularly defined effector mechanisms and can be regulated by pharmacological or genetic interventions^40,47^. Depending on the morphological appearance of dying cells, RCD can be classified as apoptosis, autophagy-dependent cell death, necroptosis, pyroptosis, ferroptosis, and others^40,47,49^. The morphological characteristics associated with apoptosis include the reduction of cellular volume (pyknosis), chromatin condensation, nuclear fragmentation, plasma membrane blebbing (with maintained membrane integrity), and cell shrinkage^40,50^. The knockdown of APE1 by RNA interference has been shown to induce apoptosis in human cancer cell lines^51^. Our data indicated that NO.0449-0145 treatment induced cell apoptosis in both A549 and NCI-H460 cells, consistent with the results reported for other APE1 inhibitors, such as APX3330, which induced apoptosis in multiple tumor cell lines^14,17^. Intriguingly, we also observed some NO.0449-0145 treated cells characterized by cytoplasmic swelling and plasma membrane bubbles, followed by plasma membrane rupture and the subsequent loss of intracellular contents. Furthermore, some treated cells displayed less swelling and produced multiple bubble-like protrusions. Hoechst33342/PI staining confirmed that the plasma membrane bubbles and then ruptures. These phenomena were consistent with pyroptosis and necroptosis^39,40,49^. Necroptosis depends on the sequential activation of RIPK3 and MLKL, whereas pyroptosis generally requires the activation of caspase-4, caspase-5, and cleaved GSDMD-N-terminal pore-forming domain (GSDMD-N)^39,40,52^. Our western blot analysis showed the increased activation of MLKL and RIPK3, as well as GSDMD-N and caspase-4, in both A549 and NCI-H460 cells treated with NO.0449-0145. The increased expression and secretion of IL-1β was observed in both A549 and NCI-H460 cells following NO.0449-0145 treatment, consistent with observations that both necroptosis and pyroptosis are associated with IL-1β secretion^40,53^. These data demonstrated that NO.0449-0145 treatment was able to induce pyroptosis and necroptosis in both NSCLC cell lines. These results indicated that APE1 repression involved several cell death pathways and highlighted the key role played by APE1 in cell proliferation and cell survival.

Thus far, chemotherapy remains the primary treatment for lung cancer patients^54^. First-line chemotherapy for advanced NSCLC is based on the backbone of platinum-doublet chemotherapy, which is associated with a median overall survival of 8–11 months^55^. However, chemotherapy often result in the development of drug resistance, leading to modest or no incremental overall survival benefit and increased toxicity. Elevated DNA repair activity has been correlated with cisplatin resistance^5^. A previous study showed that the co-incubation cells with APX3330 and cisplatin significantly decreased cell viability compared with cisplatin alone^17^. Our study showed that NO.0449-0145 treatment alone was able to overcome cisplatin resistance in A549-DDP cell and sensitize A549-DDP cells to cisplatin treatment, implying that the inhibition of APE1 by a small-molecule inhibitor may be used to overcome chemotherapeutic resistance in lung cancer treatment. These results were consistent with previous study that bifunctional APE1 inhibitor AT-101 enhances the chemosensitivity of cisplatin in NSCLC cells through inhibition of both APE1 DNA repair and redox activity^56^.

Targeted therapies have significantly improved the treatment of NSCLC in selected patient populations. NSCLC cases associated with specific genomic mutations have benefited from targeted therapies. Approximately 30–50% of NSCLC cases of east Asian descent harbor EGFR mutations, compared with 10–20% of cases among those who are not of east Asian descent^57^. The first targeted drugs for NSCLC were gefitinib and erlotinib, both of which are small-molecule TKIs that act against EGFR^58^. Several clinical trials have established the superiority of EGFR TKIs as first-line treatments in EGFR-mutated NSCLC^54,58^. However, acquired resistance develops in nearly all patients who receive EGFR-targeted TKI treatments through mechanisms that include the development of secondary EGFR mutations, pathway bypass or alternative activation processes, or histological transformations^54^. The increased expression of APE1 in EGFR‐TKI‐resistant cells implicated APE1 in the development of TKI resistance n NSCLC, suggesting that targeting APE1 may represent a strategy for overcoming the acquired resistance of NSCLC cells against TKI treatment^33,34^. We further investigated the effects of NO.0449-0145 treatment on erlotinib-resistant NCI-H1975 cells (NCI-H1975-ER). We found that NO.0449-0145 treatment induced DNA damage and cytotoxicity in erlotinib-resistant NCI-H1975-ER cells, resulting in cell death (Supplementary Fig. 10a–e). However, NO.0449-0145 treatment had no effect on the sensitivity of NCI-H1975-ER cells to erlotinib (Supplementary Fig. 10f), which suggested that NO.0449-0145 treatment did not reverse the TKI resistance of NSCLC cells. These results were consistent with previous reports that another APE1 DNA repair activity inhibitor, APE1 inhibitor III, had no effect on TKI-induced cell growth inhibition and apoptosis, whereas the APE1-redox inhibitor E3330 significantly enhanced TKI-induced cell growth inhibition and apoptosis and modulated the expression of pro-apoptotic and anti-apoptotic proteins in TKI-resistant LUAD cells^33^.

We identified a novel APE1 endonuclease inhibitor, NO.0449-0145, which was able to induce DNA damage at a low concentration, leading to cell apoptosis, pyroptosis, and necroptosis of NSCLC cells, both in vitro and in vivo. We identified the association between APE1 and NSCLC and confirmed the critical role represented by APE1 as a cancer therapy target. Our findings suggested that NO.0449-0145 has great potential for the treatment of NSCLC and other tumors.

## Materials and methods

### Cell lines and cell culture

The HELF, A549, NCI-460, and NCI-H1299 cells were obtained from the American Type Culture Collection (ATCC) (Manassas, VA, USA). The cisplatin-resistant A549/DDP cells were Tumor Cell Bank of the Chinese Academy of Medical Science and maintained in the presence of 10 μM Cisplatin (DDP) (P4394, Sigma). NCI-H1975 erlotinib-resistant cell line (NCI-H1975/ER) was purchased from Shanghai Haling Biological Technology Co., LTD (Shanghai, China) and has been detected by STR genotyping. All cells were cultured in recommended medium with 10% FBS at 37 °C in a humidified 5% CO_2_ incubator. Cells are tested for mycoplasma contamination.

### Compounds and antibodies

Fifty two kinds of small molecular compounds and CRT0044876 were purchased from Topscience Technology Limited Company (Shanghai, China). APX3330 was obtained from GLPBIO (Montclair, CA, USA). All compounds were dissolved in dimethyl sulfoxide (DMSO), and then aliquoted and stored at −20 °C. HELF was cultured in DMEM (Gbico, USA) and A549, NCI-H460, NCI-H1299 was cultured in RPMI 1640 (Gbico, USA) supplemented with 10% fetal bovine serum (Gbico, USA). The following antibodies were used: anti-53BP1 antibodies were from Santa Cruz Biotechnology (#sc-515841,Santa Cruz, CA, USA); anti-Mcl-1(#94296), Bcl-XL (#2764), BAD(#9268), BAK(#12105), Caspase-8(#8592), and γ-H_2_AX (#7631) antibodies were from Cell Signaling Technology; anti-MLKL (#19685), phospho-MLKL(#AP0949), GSDMD (#A18281), Caspase-4 (#19305) antibodies were from ABclonal Technology; anti-APE1 (#10203-1-AP) and anti-RIPK3 (#17563-1-AP) was from Proteintech; phospho-RIPK3 (#AP7443) and anti-TBB5 (tubulin beta-5 chain) (#AW1030A) antibody was from Abgent (San Diego, CA); goat anti-mouse IgG-horseradish peroxidase and goat anti-rabbit IgG-horseradish peroxidase were from Vazyme biotech co. (Nanjing, China).

### Colony-forming assay

The colony-forming assay was performed with different kinds of cells as described previously^42^. Briefly, about 500 cells were seeded in a six-well plate and incubated for ~15 days at 37 °C. The cells were then washed with PBS and stained with 0.05% crystal violet. Stained plates were washed and dried prior to scoring the colonies.

### AP site cleavage assay

The AP site cleavage assay was performed as previously described, with slight modification^26^. Briefly, the substrate 5’-FAM-GCCCCCFGGGGACGTACGATATCCCGCTCC-3’ and its complementary sequence 3’-Q-CGGGGGCCCCCTGCATGCTATAGGGCGAGG-5’ were denatured at 95 °C for 10 min, annealed at room temperature, and stored at −20 °C until use. Purified APE1 (1.5 ng) was incubated with the substrate in reaction buffer (1 mM MgCl_2_, 50 mM Tris–HCl [pH 8.0], 50 mM NaCl, and 2 mM DTT) at 37 °C for 20 min. Small molecular compounds (5 µM) or an equal volume DMSO vehicle control were added to the reaction buffer. Fluorescence was captured and recorded at 37 °C for 20 min using a microplate reader (Inifit2000, Tecan) at 495 nM excitation and 530 nM emission. Data analysis was performed by Prism 6 software. Each experiment was repeated three independent times.

### Circular dichroism spectroscopy analysis

The combining ability of NO.0449-0145 to APE1 was studied using Circular Dichroism (CD) analysis. APE1 was incubated with NO.0449-0145 in reaction buffer (1 mM MgCl2, 50 mM Tris–HCl [pH 8.0], 50 mM NaCl, and 2 mM DTT) at 37 °C for 30 min and spectra were scanned at a wavelength of 180–280 nm using 0.001 cm path length quartz cell on circular dichroism (Chirascan, Applied Photophysics). The spectra of reaction buffer alone were subtracted for the experimental data. CD spectra of APE1 incubated with DMSO was served as the negative control. The secondary structure for APE1 with NO.0449-0145 or DMSO was analyzed using GraphPad Prism.

### Immunofluorescence assay

Cells were grown on round cover-glass in 12-well plates to 60% confluence. After 24 h, cells were treated with different concentrations of NO.0449-0145 or DMSO, which was served as control for 24 h. And then cells were washed with PBS for three times and added 4% PFA at room temperature (RT) for 15 min. After permeabilized with 0.2% Triton X-100, cells were incubate with primary antibodies at 4 °C overnight and then washed with PBS for three times following incubation with fluorescent secondary antibodies at RT for one and half hours. Then, DAPI staining were performed at 37 °C for 15 min and cells were sealed with antifluorescence quenching seal tablet and the cover-glass. For tumor samples, the tissues were excised and fixed in 10% formalin and made into paraffin embedded sections. The paraffin sections were heated at 65 °C for dewaxing. Immunofluorescence staining for γ-H2AX and 53BP1 was performed using corresponding antibodies. The fluorescence microscope was used to detect the fluorescence intensity.

### MTT assay

Cells were seeded in 96-wells plates at 3.0 × 10^3^ /well (in triplicate) and hatched overnight. Cells were treated with the increasing concentrations of NO.0449-0145 for 48 h. The culture medium containing NO.0449-0145 was sucked out, and then the medium containing 20 μL of MTT (5 mg/ml) per well was added. After four hours, the medium including MTT was discard and 100 μL of DMSO was added. Cellular survival was calculated by reading the absorbance of the formazan at 570 nm in enzyme-labelling instrument (Inifit2000, TECAN).

### Apoptosis analysis by flow cytometry

Cells were seeded in six-well plates at a density of 2 × 10^5^ cells/well (in triplicate) and grown overnight. And then cells were treated with NO.0449-0145 in increasing concentrations (0.1, 0.2, 0.3 μM) and DMSO as control. After 24 h, cells were harvested and then incubated with PI and Annexin V-FITC using an apoptosis detection kit (KGA107) following the instructions. Finally, cells were tested in flow cytometry (Becton Dickinson, FACS Verse, USA). FlowJo7.6 software was used for data analysis.

### Mitochondrial membrane potential (MMP) analysis

A549 or NCI-H460 cells, which were seeded in six-well plates and grown overnight, were treated with the increasing concentrations of NO.0449-0145 for 24 h. Mitochondrial membrane potential (MMP) analysis was assessed by JC-1 staining according to the instructions of JC-1 Apoptosis Detection Kit (KGA601-KGA604, Keygen Biotech, Nanjing, China). Briefly, cells were harvested and incubated with JC-1 staining buffer for 30 min. The intensity of JC-1 staining was measured by flow cytometry at the excitation wavelength of 488 nm and the emission filter of 530 nm (BD Biosciences FACS Calibur, USA).

### Tumor xenograft model

All animal experiments were performed according to procedures approved by the Laboratory Animal Care Committee at Nanjing Normal University, in accordance with the National Institutes of Health Guide for the Care and Use of Laboratory Animals. Female BALB/c nude mice (purchased from GemPharmatech Co., Ltd., Nanjing, China) were housed and fed with a standard diet throughout the course of the experiment. Six-week-old nude mice were injected with 100 µl serum-free medium suspension containing NCI-H460 cells (1 × 10^6^). Approximately 5–7 days later, when the tumors reached 50–100 mm^3^, the mice were randomized into four groups. Mice were intraperitoneally administered either NO.0449-0145 (3.125, 6.25, or 12.5 mg/kg) or vehicle (saline) control every other day for 3 weeks. Tumor sizes were measured using caliper every three days, and tumor volumes (mm^3^) were calculated as width × height × length/2. Mouse body weights were recorded every three days. At the end of the experiment, all mice were sacrificed by cervical dislocation. The tumors were collected, photographed, weighed, and processed for paraffin sections.

### Immunochemistry analysis

Lung cancer tissue microarray (TMA) slides (LC1005a) were purchased from Avilabio (Xian, China). This slide contains 45 cases of non-small cell carcinoma (26 squamous cell carcinoma, 18 adenocarcinoma, and 1 adenosquamous carcinoma) and matched cancer adjacent lung tissues, and five normal lung tissues with duplicate cores per case. Patients provided informed consent at the time of surgery. Mouse xenograft tumor tissues were excised and fixed in 10% formalin and made into paraffin embedded sections. For immunochemistry analysis, sections were deparaffinised, hydrated, and boiled for 10 min in 10 mM citrate buffer (pH 6.0), followed by cooling for 20 min at RT. Sequential blocking (3% BSA) for 1 h each was used to prevent unspecific antibody binding. Staining was performed for Ki67 and else was performed using corresponding antibodies overnight at 4 °C. Subsequently, sections employ a biotinylated secondary antibody, streptavidin-HRP conjugates and DAB Kit (Invitrogen) as the chromogenic substrate. The sections were then counterstained with haematoxylin.

### Electrophoretic mobility shift assay (EMSA)

EMSA was adapted to measure APE1-redox activity as previously described^59^. The purified Ape1 protein was reduced with 0.2 mM DTT at 37 °C for 10 min and diluted in PBS buffer to final concentrations of 0.2 μg/μl. Reduced APE1 (2 μl) were incubated with purified AP-1 in EMSA reaction buffer (50 mM NaCl, 5 mM MgCl_2_, 10 mM Tris–HCl pH 7.5) for 20 min at 37 °C. FAM-labelled double-stranded DNA (5’-FAM-CGCTTGATGACTCAGCCGGAA-3’) (0.1 pmol) was added into the reaction solution (total 20 μl) and incubated for another 10 min at 90 °C. The samples were subjected to 5% nondenaturing polyacrylamide gels (TBE-PAGE) at 4 °C, 100 V for 50 min and then scanned with an Odyssey imager (LI-COR).

### Statistical analysis

All experiments in the study were conducted in triplicates. The results are presented as mean ± SD. The statistical evaluation was performed by unpaired two-tailed Student’s *t*-test between two groups. The statistical significance was cognizance of *p*-values < 0.05 and statistical analyses were analyzing using GraphPad Prism 6 (GraphPad Software, San Diego, CA, USA).

## Supplementary information

Supplementary tables and Figures
