# Supplementary material for: Small-molecule inhibition of APE1 induces apoptosis, pyroptosis, and necroptosis in non-small cell lung cancer
Source: Cell Death Dis. 2021 May 18;12(6):503. doi: 10.1038/s41419-021-03804-7 (PMC8131371; doi:10.1038/s41419-021-03804-7)
Supplement: Supplementary file 1 — Supplementary tables and Figures [file 41419_2021_3804_MOESM1_ESM.docx]

**Small-molecule inhibition of APE1 induces apoptosis, pyroptosis, and necroptosis in non-small cell lung cancer**

Kaili Long^§^, Lili Gu^§^, Lulu Li^§^, Ziyu Zhang, Enjie Li, Yilan Zhang, Lingfeng He, Feiyan Pan, Zhigang Guo*, Zhigang Hu*

^1^Jiangsu Key Laboratory for Molecular and Medical Biotechnology, College of Life Sciences, Nanjing Normal University, Nanjing210023, China

**Supplementary Information**

The following file contains supplementary material for the paper “Small-molecule inhibition of APE1 induces apoptosis, pyroptosis, and necroptosis in non-small cell lung cancer”.

This file is composed of:

 Supplementary tables (3 tables)

 Supplementary figures and relative supplementary figure legends (10 figures)

**Supplementary table 1.** **52 small molecular compounds as potential APE1 inhibitors**

| Number | Name | Number | Name | Number | Name |
| --- | --- | --- | --- | --- | --- |
| 1 | 1554-0035 | 18 | 5632-0002 | 35 | N027-0014 |
| 2 | N039-0006 | 19 | Y041-5136 | 36 | N016-0072 |
| 3 | N006-0008 | 20 | V029-4535 | 37 | 0564-0012 |
| 4 | N039-0038 | 21 | D158-0309 | 38 | 0195-0043 |
| 5 | N040-0008 | 22 | 1079-1472 | 39 | Y020-5588 |
| 6 | 0080-0014 | 23 | 8018-2981 | 40 | 8010-7744 |
| 7 | Y041-5135 | 24 | 0350-0159 | 41 | F248-0087 |
| 8 | 0449-0145 | 25 | N008-0008 | 42 | N014-0003 |
| 9 | Y041-5137 | 26 | 1496-0846 | 43 | 1649-0099 |
| 10 | N001-0008 | 27 | 8018-3006 | 44 | 8011-1606 |
| 11 | 2061-0044 | 28 | 4826-0360 | 45 | 8018-0020 |
| 12 | 0083-0101 | 29 | 0108-0001 | 46 | 8018-2999 |
| 13 | N014-0006 | 30 | 8019-8689 | 47 | Y020-8461 |
| 14 | 4554-4707 | 31 | 0157-0007 | 48 | 4896-0596 |
| 15 | V028-1508 | 32 | 6927-0944 | 49 | 8019-6463 |
| 16 | 2159-4171 | 33 | 8640-0051 | 50 | Y030-1255 |
| 17 | 8018-6926 | 34 | N039-0028 | 51 | 0270-0072 |
|  |  |  |  | 52 | 6232-2549 |

| Number | Inhibitors | Enzyme Activity (%) |
| --- | --- | --- |
| Positive Control | CRT0044876 | 36 |
| 5 | N040-0008 | 29 |
| 8 | 0449-0145 | 24 |
| 12 | 0083-0101 | 24 |
| 16 | 2159-4171 | 25 |
| 23 | 8018-2981 | 26 |
| 36 | N016-0072 | 34 |
| 46 | 8018-2999 | 35 |
| 50 | Y030-1255 | 35 |

**Supplementary table 2. Eight candidate small molecule inhibitors of APE1**

**Supplementary table 3. Half-maximal inhibitory concentration (IC50) among the eight tested compounds.**

| Number | Inhibitors | IC50(μM) |
| --- | --- | --- |
| CON | CRT0044876 | >>50 |
| 5 | N040-0008 | >>50 |
| 8 | 0449-0145 | 0.1068 |
| 12 | 0083-0101 | >>50 |
| 16 | 2159-4171 | >>50 |
| 23 | 8018-2981 | >>50 |
| 36 | N016-0072 | >>50 |
| 46 | 8018-2999 | >>50 |
| 50 | Y030-1255 | >>50 |

**
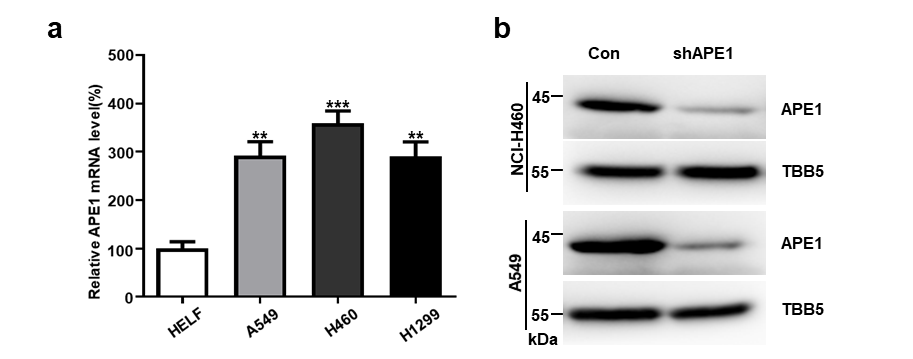
**

**Supplementary Figure 1.** The expression of APE1 in NCI-H460 and A549 cells.

**a** APE1 mRNA levels in normal lung HELF cell, NSCLC cell lines A549, NCI-H460 and NCI-H1299. **b** Western blot assay in control or APE1-KD NCI-H460 and A549 cells. **P<0.01; ***p < 0.001.

**
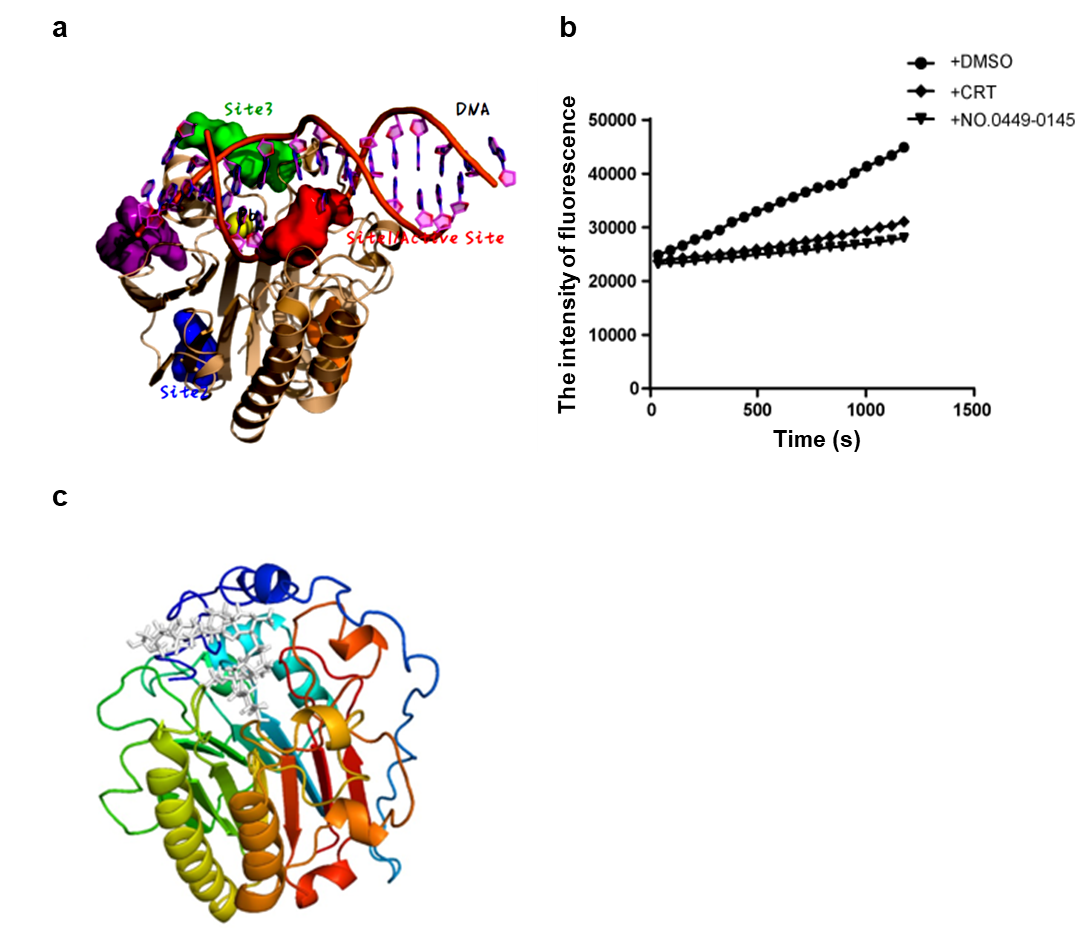
**

**Supplementary Fig 2.** Molecular structure of the inhibitor. **a** The four best druggable sites of the APE1 protein. **b** The AP site cleavage assay when adding negative control DMSO or effective inhibitors. **c** The structures of APE1 and NO.0449-0145 were analyzed by molecular simulation.

**
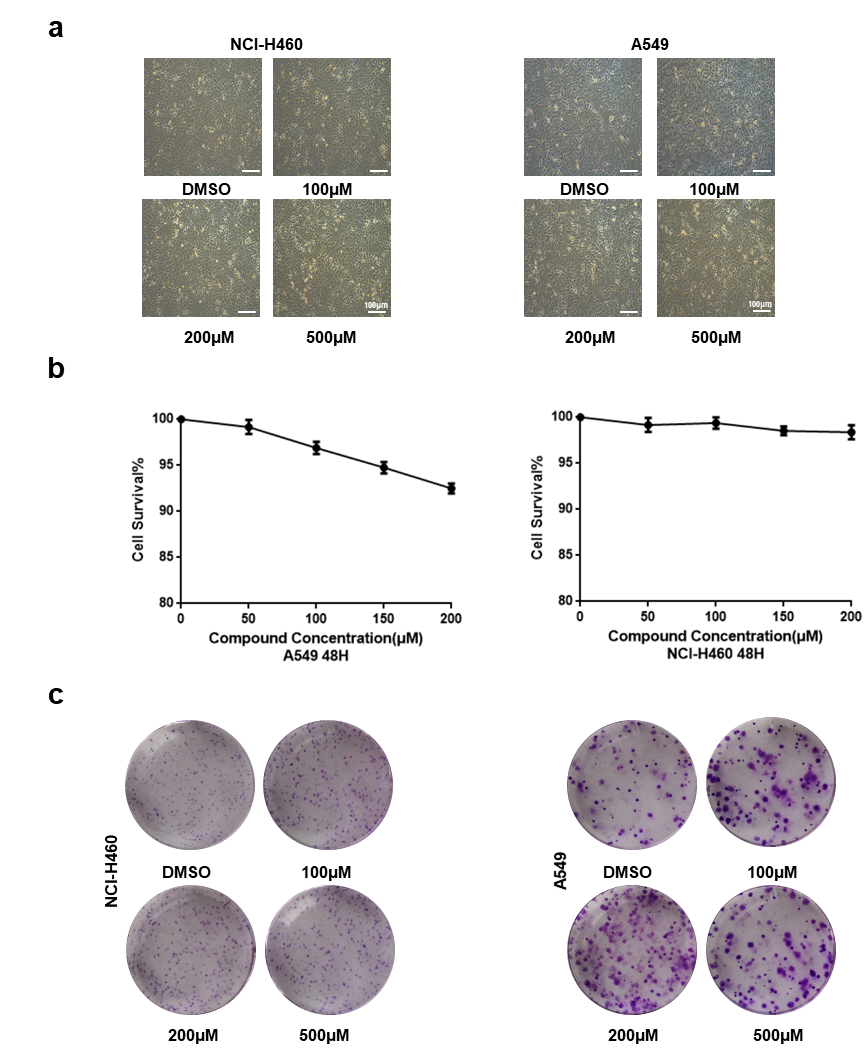
**

**Supplementary Fig 3.** Inhibition APE1 by CRT0044876 showed no cytotoxic effects in lung cancer cells at high concentrations. **a** Morphological analysis of NCI-H460 and A549 cells with different dose of CRT0044876 treatment. **b** Cell survival assay in NCI-H460 and A549 cells treated with CRT0044876 for 48 h. **c** Colony-formation assay with different dose of CRT0044876 treatment in A549 and NCI-H460 cells.


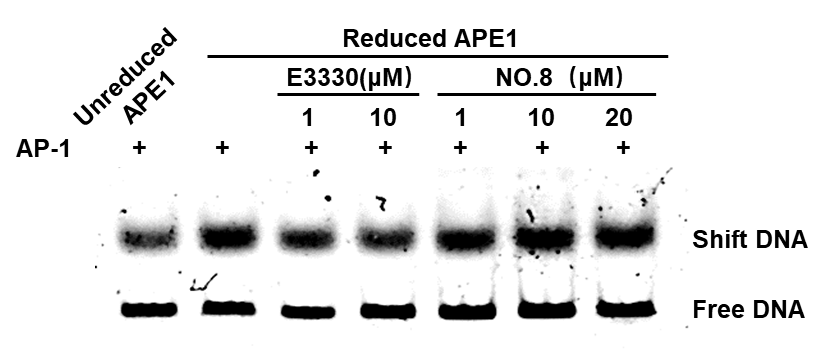


**Supplementary Figure 4.** Effect of NO.0449-0145 on APE1 redox activity by EMSA with purified AP-1 and APE-1 proteins. A double-stranded DNA carrying an AP-1 binding motif was mixed with purified AP-1. Purified APE1 or reduced APE1 (treated with 0.2 mM DTT) were added to the reaction. AP-1 binding DNA was examined by EMSA.

**
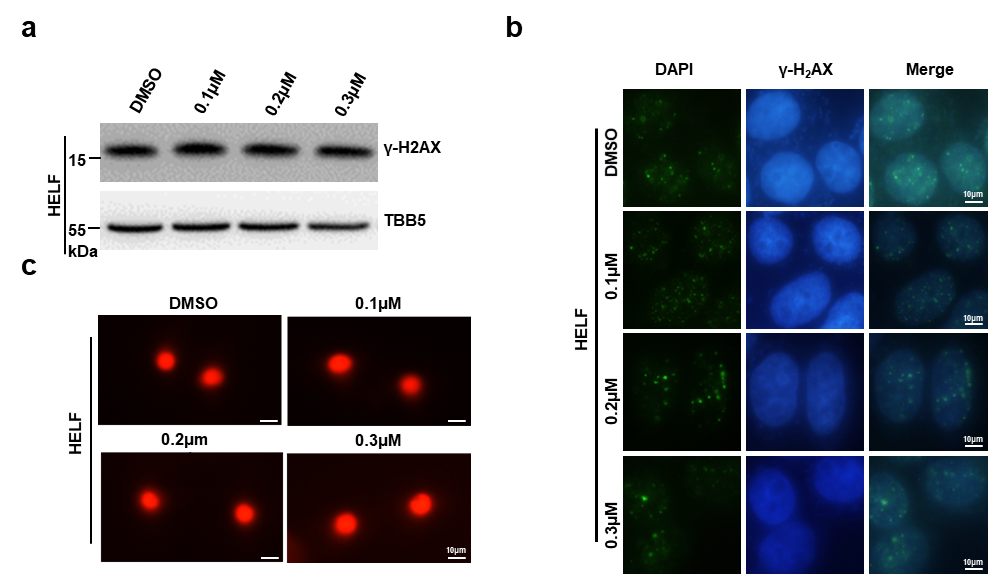
**

**Supplementary Fig 5.** NO.0449-0145 treatment has no effect on the accumulation of DNA damage in HELF cell. **a** Western blot assay to determine γ-H2AX levels in HELF cell with different dose of NO.0449-0145 treatments. **b** Immunofluorescence staining of γ-H2AX in HELF cell. **c** Comet assay of HELF cell treated with or without NO.0449-0145.

**
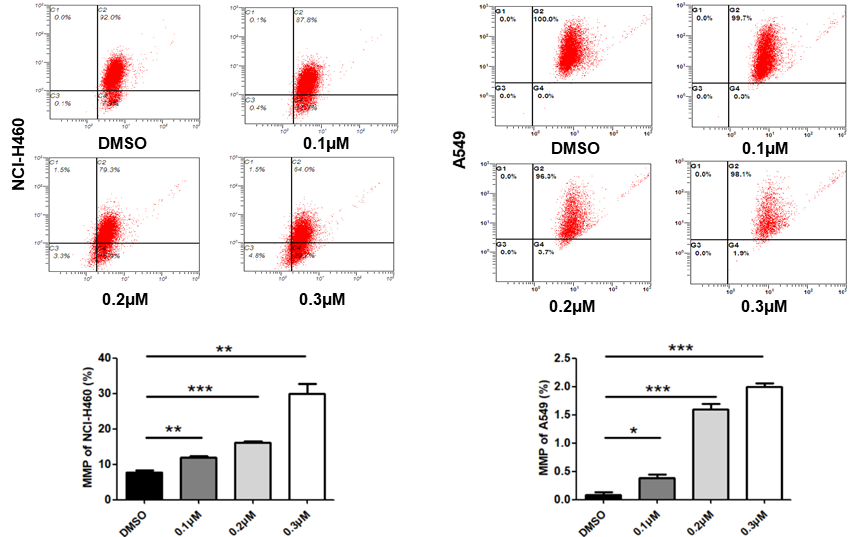
**

**Supplementary Fig 6.** JC-1 staining and flow cytometry shows different change in the MMP in NCI-H460 and A549 cells treated with the same dose of NO.0449-0145. Two-tailed Student’s t-test, *P < 0.05; **P<0.01; ***p < 0.001.


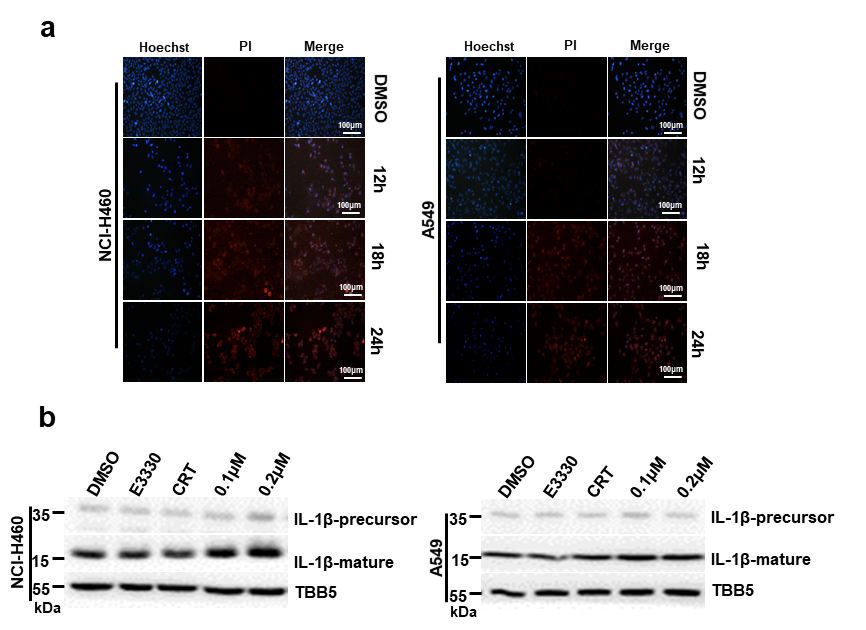


**Supplementary Fig 7. a** Representative Hoechst/PI fluorescence images of cells with time gradient treatment(12h,18h,24h). **b** Western blot assay tests the protein expression of IL-1β.


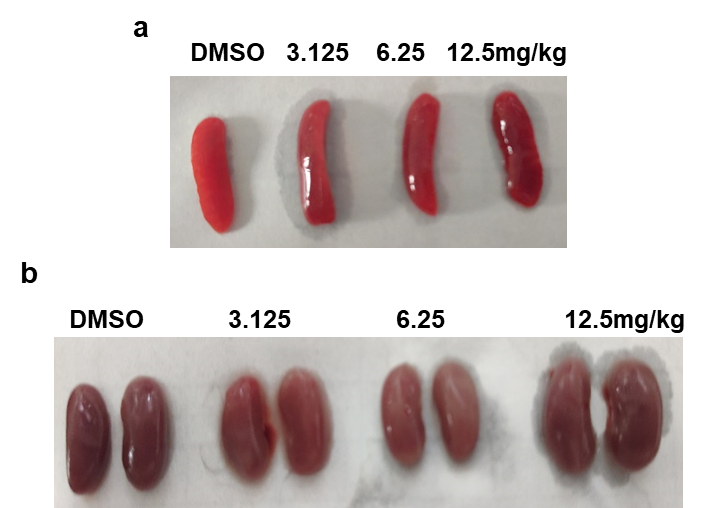


**Supplementary Figure 8.** Different doses of NO.0449-0145 used in our study do not affect the morphology of **a** Spleen and **b** Kidney in different treated mice.

**
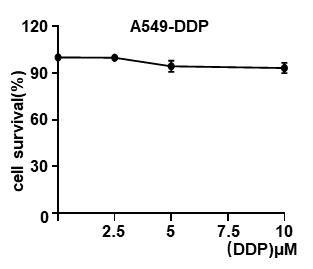
**

**Supplementary Figure 9.** Cell survival assay in A549-DDP cells treated with cisplatin for 48 h**.**

**
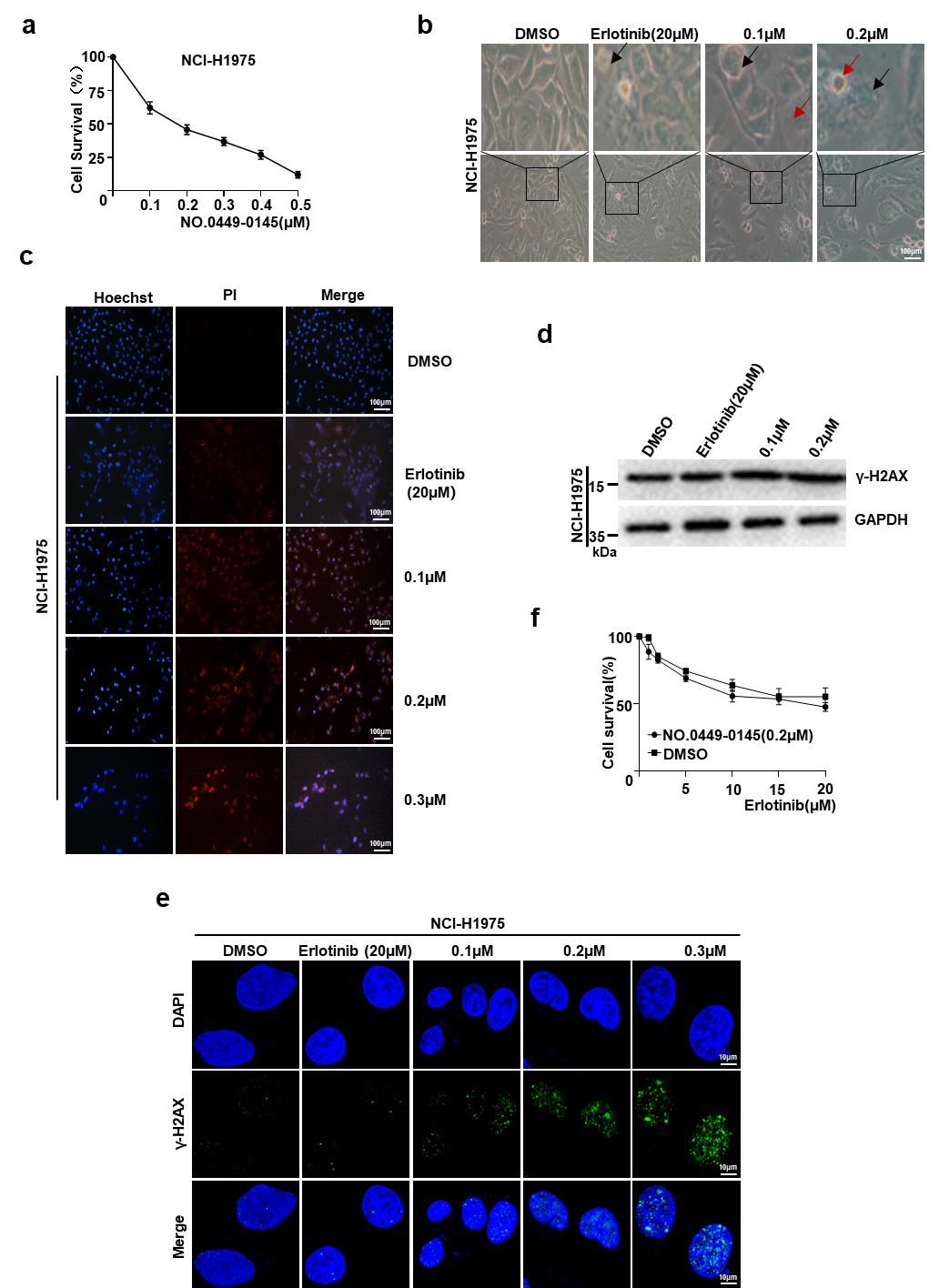
**

**Supplementary Figure 10.** NO.0449-0145 overcomes the erlotinib-resistant NCI-H1975 cells. **a** Cell survival assay in NCI-H1975-ER cells treated with NO.0449-0145 for 48 h. **b** Morphological analysis of different treated NCI-H1975-ER cells. **c** Representative Hoechst/PI fluorescence images of NCI-H1975-ER cells with different treatment. **d** Western blot assay tests the protein expression of γ-H2AX. **e** Immunofluorescence staining of γ-H2AX in NCI-H1975-ER cells with different treatment. **f** Cell survival assay in NCI-H1975-ER cells in erlotinib with or without NO.0449-0145 for 48h.
